# Trends in sustainable dietary patterns in United States adults, 2007-2018

**DOI:** 10.4178/epih.e2025045

**Published:** 2025-08-18

**Authors:** Sukyoung Jung, Heather A. Young, Barbara H. Braffett, Samuel J. Simmens, Eunice Hong Lim Lee, Cynthia L. Ogden

**Affiliations:** 1 Department of Healthcare Policy Research, Korea Institute for Health and Social Affairs, Sejong, Korea; 2 Department of Epidemiology, Milken Institute School of Public Health, The George Washington University, Washington, DC, USA; 3 Department of Biostatistics and Bioinformatics, Milken Institute School of Public Health, The George Washington University, Washington, DC, USA

**Keywords:** Sustainable diet, Dietary sustainability, Sustainable diet index, Dietary patterns, Trend, National Health and Nutrition Examination Survey

## Abstract

**OBJECTIVES:**

Adopting sustainable diets is essential for improving both human and planetary health, and such diets should be evaluated from a multidimensional perspective. We characterized trends in sustainable dietary patterns, quantified by the sustainable diet index for United States (SDI-US) adults, along with trends in diet quality, diet-related environmental impacts, food affordability, and food practices.

**METHODS:**

This study analyzed data from the National Health and Nutrition Examination Survey (2007-2018) for adults aged ≥20 years (n=25,543). The SDI-US (range, 4-20 points), with higher scores indicating more sustainable diets, was calculated using 24-hour dietary recall data and responses to consumer and dietary behavior questionnaires. Mean total SDI-US scores, sub-indices, and 12 individual indicators were estimated for each survey cycle. Trends were assessed using orthogonal polynomial contrasts in regression models.

**RESULTS:**

From 2007 to 2018, total SDI-US scores showed no significant overall trend (overall mean, 13.1). Nutritional and socio-cultural indicators remained relatively stable, whereas the economic indicator (food expenditures) worsened from 21.4% to 26.4% (p<0.05, linear trend) between 2007-2008 and 2017-2018. Environmental impacts initially worsened between 2007-2008 and 2013-2014 but improved through 2017-2018 (all p<0.05, quadratic trend). When stratified by age (p for interaction <0.001), a slight decline in SDI-US was observed among adults aged ≥60 years (14.1 to 13.9, p<0.001).

**CONCLUSIONS:**

From 2007 to 2018, total SDI-US scores largely remained unchanged, although declines occurred among adults ≥60 years and scores remained lower among adults aged 20-39 years. Ongoing monitoring and coordinated improvements across all dimensions are needed to advance sustainable diets in all age groups.

## GRAPHICAL ABSTRACT

[Fig f2-epih-47-e2025045]

## Key Message

Among 25,543 US adults aged 20 years and older, overall sustainable diet index (SDI-US) scores showed no significant change between 2007 and 2018. While food expenditure share worsened and environmental indicators recently improved, declines were observed among older adults (≥60 years) and lower scores persisted among younger adults (20-39 years). These findings highlight the need for ongoing monitoring and tailored strategies to promote sustainable diets across age groups.

## INTRODUCTION

In 2005, the Giessen Declaration emphasized that new nutrition science should address not only the biological dimension but also environmental and social dimensions to confront global challenges [1]. The Food and Agriculture Organization of the United Nations (FAO) defines sustainable diets as “diets with low environmental impacts which contribute to food and nutritional security and to healthy life for present and future generations” and “are protective and respectful of biodiversity and ecosystems, culturally acceptable, accessible, economically fair and affordable; nutritionally adequate, safe, and healthy; while optimizing natural and human resources” [2]. Recent global initiatives to achieve sustainability include transforming current food systems by changing individual dietary practices and advancing technological and organizational innovations [3,4].

Based on the FAO definition, sustainable dietary patterns should be assessed through a multidimensional framework that holistically considers 4 key diet dimensions: nutritional, environmental, economic, and socio-cultural [2]. Within this framework, the sustainable diet index for United States (SDI-US) adults was recently developed and validated in a United States cohort [5]. The SDI-US comprises 4 sub-indices of sustainability, each including 12 indicators, with higher scores indicating greater adherence to sustainable diets (score range, 4 to 20) [5]. Similar indices have been examined in France [6], Spain [7], Mexico [8], and Ghana [9]. Greater adherence to sustainable diets has been inversely associated with the risk of obesity [10,11], cancer [12], cardiovascular disease [12], and all-cause mortality [7,13].

Global and national policies increasingly reflect a shift toward sustainable diets. For example, the Healthy Diets Monitoring Initiative was launched in 2022 through a partnership among the FAO, United Nations Children’s Fund, and World Health Organization to support global progress toward diets that benefit both people and the planet [14]. In the United States, the 2015 Dietary Guidelines Advisory Committee examined for the first time the scientific links between dietary patterns and environmental sustainability [15]. Additionally, a substantial proportion of United States adults have recently reduced their consumption of red and processed meat, citing health and cost as primary motivations, with environmental considerations playing an increasing role in food choices [16].

Despite these positive developments, no study has assessed whether sustainable dietary patterns and their individual indicators—including diet-related environmental impacts, food affordability, and food practices—have changed over time. Understanding such temporal trends is important for evaluating progress in dietary sustainability. Therefore, we examined changes in total SDI-US and in each indicator score among United States adults from 2007 to 2018. We aimed to determine whether dietary sustainability in the United States has shifted over a 12-year period in the context of the broader global movement toward sustainable diets. To address this question, we analyzed data from 6 consecutive cycles of the National Health and Nutrition Examination Survey (NHANES) spanning 2007-2008 to 2017-2018.

## MATERIALS AND METHODS

### Study population

NHANES is an ongoing, cross-sectional, nationally representative survey designed to assess the health and nutritional status of the non-institutionalized civilian Unite States population [17]. Since 1999, NHANES has continuously collected data and released it publicly in 2-year cycles. To ensure representativeness, NHANES employs a complex, multistage probability sampling design [18]. Data collection occurs year-round and includes a household interview; a visit to a mobile examination center (MEC) for standardized physical examinations, laboratory tests, health interviews, and dietary assessments (including 24-hour dietary recalls conducted by trained staff); and post-MEC follow-up [19]. Details of NHANES data collection procedures are provided in Supplementary Material 1.

Among 34,770 adults aged 20 years and older, we excluded participants who were pregnant or breastfeeding (n=580); did not complete the first 24-hour dietary recall (n=3,944); lacked information on serum vitamin D [25(OH)D] levels (n=2,058), SDI-US score components (family income, food expenditures, ready-made product use behaviors) (n=2,628), or education level (n=17). The final analytic sample comprised 25,543 adults (Supplementary Material 2).

### Assessment of outcome: sustainable diet index for United States

The primary outcome was adherence to sustainable diets, assessed by the SDI-US score. The SDI-US includes 12 indicators grouped into 4 sub-indices: the nutritional sub-index (2 indicators) reflecting diet quality; the environmental sub-index (6 indicators) capturing environmental impacts; the economic sub-index (1 indicator) measuring food affordability; and the socio-cultural sub-index (3 indicators) addressing food practices, including ready-to-eat product use (Supplementary Material 3). The details of the SDI-US development and validation are available in previous articles [5,11,13]. A previous validation study demonstrated that the SDI-US distinguishes dietary patterns across demographic subgroups with known differences in diet quality [5]. While scores tended to cluster near the mean, the index displayed a reasonably normal distribution without substantial ceiling or floor effects, and the 5th to 95th percentile range (8.8-17.2) indicated meaningful variation across the population. Briefly, the Nutrient-Rich Foods (NRF) 9.3 index was calculated using the formula described in earlier work [20,21]:


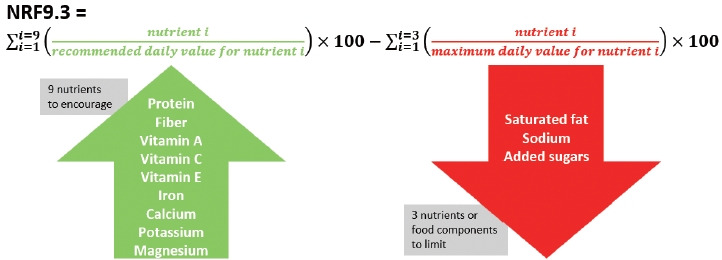


The mean nutrient adequacy ratio (MAR) was computed as the average of nutrient adequacy ratios (NARs) for 12 nutrients: vitamin A (μg RAE), thiamin (mg), vitamin B6 (mg), folate (μg DFE), vitamin C (mg), vitamin E as alpha-tocopherol (mg), calcium (mg), copper (mg), iron (mg), magnesium (mg), zinc (mg) [22], and serum 25(OH)D (nmol/L). All nutrients in the NAR calculation were energy-adjusted using the residual method [23] to correct for measurement error due to underreporting [24,25] and to minimize variation in intake related to age, sex, body size, and metabolic efficiency [26]. The NAR was calculated as the ratio of the amount of a given nutrient consumed to its recommended dietary allowance [27] or recommended threshold for deficiency risk in the case of serum 25(OH)D (<50 nmol/L) [28].


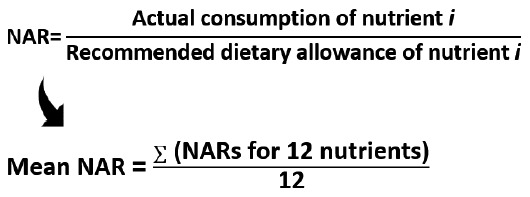


Worked examples of the calculation process for each nutritional indicator are provided in Supplementary Material 4.

Environmental impacts were estimated using a predeveloped database derived from a meta-analysis of 1,530 publications, covering 43 food groups [29]. Six environmental impact measures were calculated: freshwater withdrawal (L), stress-weighted freshwater withdrawal (L), acidifying emissions (g SO₂ eq), eutrophication emissions (g PO₄³^-^ eq), greenhouse gas emissions (GHGE; kg CO₂ eq), and land use (m²). Each food item from the 24-hour dietary recalls (NHANES 2007-2008 through 2017-2018) was hand-coded and matched to the database food groups to derive the corresponding environmental impact estimates.

Total monthly food expenditures were calculated as the sum of spending over the past 30 days on food purchased from supermarkets or grocery stores, other retailers, dining out, and takeout or delivery. This amount was annualized by multiplying by 12, and food affordability was expressed as the percentage of total annual food expenditures relative to total annual family income.

Food practices were assessed based on the reported frequency of meals prepared away from home, meals from fast-food or pizza outlets, ready-to-eat foods, and frozen meals or frozen pizzas during the past 7 days or 30 days.

For scoring, all indicators except socio-cultural ones were categorized into quintiles and assigned scores from 1 to 5, with higher scores reflecting better nutrition, lower environmental impact, or greater affordability. For socio-cultural indicators, participants reporting zero frequency received a score of 5; those in the first quartile were assigned a score of 4, and those in the fourth quartile a score of 1. Sub-index scores were the averages of indicator scores within each sub-index, and the total SDI-US score was the sum of all sub-index scores (range, 4 to 20).

### Statistical analysis

The general characteristics of the study population, including age group, sex, race/Hispanic origin, education level, and household size, were described as the unweighted number of participants (sample size) and weighted proportions by survey cycle. Mean SDI-US scores, sub-index scores, and individual indicator values were estimated, and the statistical significance of linear and quadratic trends over time was tested using survey cycle modeled as an orthogonal polynomial in linear regression models. Multivariable models were adjusted for age, sex, race/Hispanic origin, education level, and household size. Potential interactions were evaluated individually by including an interaction term between survey cycle and each covariate in the regression models. Subgroup analyses were conducted by age (20-39, 40-59, ≥60 years), sex (male, female), race/Hispanic origin (total Hispanic, non-Hispanic White, non-Hispanic Black, other), education level (less than high school graduate, high school graduate or equivalent, some college or above), and household size (single-person vs. multi-person households). Sensitivity analyses were performed as described in Supplementary Material 5.

All analyses incorporated dietary Day 1 sample weights to account for differential probabilities of selection, non-response, non-coverage, and day of the week, along with survey design variables for clustering and stratification. Statistical analyses were performed using SAS version 9.4 (SAS Institute Inc., Cary, NC, USA) with survey analysis procedures, and an α level of 0.05 was considered statistically significant.

### Ethics statement

The study protocols of NHANES were approved by the National Center for Health Statistics Ethics Review Board (https://www.cdc.gov/nchs/nhanes/irba98.htm). This study was deemed exempt by the George Washington University Institutional Review Board because we only used publicly available data without identifiable information.

## RESULTS

### Participants’ characteristics

From 2007-2008 to 2017-2018, the proportion of adults aged ≥60 years increased from 24.6% to 29.7%, while the proportion of non-Hispanic White adults declined from 72.5% to 64.2%. The proportion of adults with at least some college education rose from 55.4% to 61.0%, whereas the proportion of single-person households decreased from 14.1% to 12.9% (Table 1). Over the same period, although changes were modest, there were linear increases in egg and nut consumption and linear decreases in total fruit consumption. Whole grain intake increased through 2011-2012 but declined thereafter through 2017-2018. Total dairy consumption rose until 2009-2010, followed by a decline through 2017-2018. Intake of refined grains, total vegetables, red meat, and added sugars showed little to no change between 2007 and 2018 (Supplementary Material 6).

### Total sustainable diet index for United States adults, sub-index, indicator scores trends over time

From 2007 to 2018, there was no significant overall trend in total SDI-US, with a mean score of 13.3 in 2017-2018. While certain sub-index scores exhibited statistically significant linear or quadratic trends, the changes in weighted means over time were minimal (Table 2). The environmental sub-index score declined slightly in 2009-2010, remained stable for several cycles, and then increased gradually from 2015-2016 to 2017-2018 (p<0.001, quadratic trend). In contrast, the economic sub-index score showed a small but consistent decline over the study period (p<0.05, linear trend).

Trends in SDI-US indicator values between 2007 and 2018 are shown in Table 3. MAR increased between 2007-2008 and 2009-2010 but subsequently declined to values similar to those in 2007-2008 (p=0.01, quadratic trend). For environmental impact indicators, water footprint rose until 2013-2014 and then declined through 2017-2018 (p<0.001, quadratic trend). Nitrogen footprint and GHGE increased from 2007-2008 to 2009-2010 and then decreased steadily through 2017-2018 (both p<0.001, quadratic trend). Land use decreased from 10.2 m² in 2007-2008 to 8.9 m² in 2017-2018 (p=0.003, linear trend). For economic indicators, the share of household budget spent on food increased from 21.4% in 2007-2008 to 26.4% in 2017-2018 (p<0.001, linear trend). Among socio-cultural indicators, the frequency of ready-to-eat product consumption rose from 1.9 times in 2007-2008 to 2.2 times in 2017-2018 (p=0.001, linear trend). The frequency of frozen meal consumption increased through 2013-2014 and then declined through 2017-2018 (p=0.02, quadratic trend).

### Subgroup and sensitivity analysis

**Figure 1.**
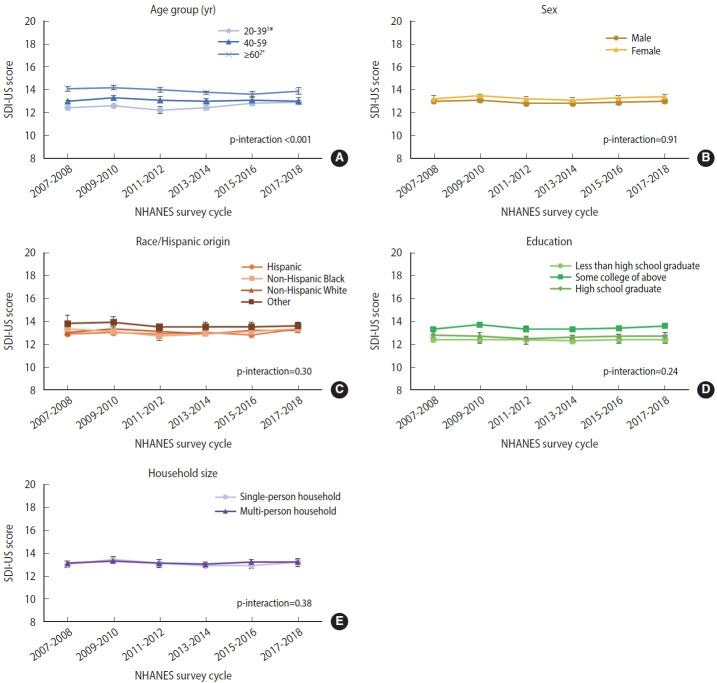
Trends in sustainable diet index for United States (SDI-US) score among population subgroups by National Health and Nutrition Examination Survey (NHANES) cycle, United States adults, 2007-2018. Estimated mean and 95% confidence interval were obtained using the linear regression model and weighted. p-values for trends were estimated with the survey cycles modeled as an orthogonal polynomial. Multivariable models were adjusted for age (years), sex (male or female), race/Hispanic origin (total Hispanic, non-Hispanic White, non-Hispanic Black, or other), education level (less than high school, high school graduate or general educational development, and some college or above), and household size (number of individuals) except the corresponding subgroup variates. p-values for interactions were determined by including the cross-product term of the SDI-US and each subgroup factor. ^1^Using quadratic trend. ^2^Using liner trend. *p<0.05.

Among demographic factors, significant heterogeneity in trends was observed by age (p<0.001 for interaction). Stratified analyses indicated temporal trends in SDI-US scores among participants aged 20-39 years and those aged ≥60 years; however, the magnitude of change was modest. For adults aged 20-39 years, the score declined from 12.4 in 2007-2008 to 12.2 in 2011-2012, then increased to 12.9 in 2017-2018 (p=0.02, quadratic trend). Among those aged ≥60 years, the score declined from 14.1 in 2007-2008 to 13.9 in 2017-2018 (p<0.001, linear trend). No significant heterogeneity was observed for trends by sex, race/Hispanic origin, education level, or household size (Figure 1).

Sensitivity analyses yielded similar findings when the Healthy Eating Index was used instead of NRF 9.3, when freshwater use was excluded, when food security level was included, and when eating together was considered (Supplementary Material 7).

## DISCUSSION

The overall SDI-US score was 13 (possible range, 4 to 20). Between 2007 and 2018, there was no significant trend in total SDI-US. While nutritional quality and food practices showed little change, food affordability worsened, and diet-related environmental impacts improved over this period. When stratified by age, a significant downward trend was observed among adults aged ≥60 years, indicating worsening dietary sustainability in this age group.

This study, using NHANES data, is the first to examine temporal trends in adherence to sustainable diets as measured by the SDI-US. Direct comparisons with prior research are limited, as few studies have investigated multidimensional sustainable diets in Unite States adults over time. Notably, adherence to sustainable diets remained largely unchanged over 12 years. This stability may reflect improvements in the environmental sub-index being offset by declines in the economic sub-index. These findings underscore that examining only 1 or 2 dimensions of sustainability risks overlooking the broader complexity of dietary patterns. A multidisciplinary approach is therefore needed—one that fosters individual awareness, knowledge, and behaviors related to sustainable diets; expands research on multidimensional measures to inform public health and policy; and actively engages policymakers in addressing sustainability challenges [30].

Although total SDI-US scores showed no significant trend, all diet-related environmental impact measures were lower in 2017-2018 compared with 2007-2008. In line with our results, a previous NHANES-based study found that GHGE from Unite States adult diets declined from 4.02 kg CO₂ eq per capita per day in 2003-2004 to 2.45 kg CO₂ eq per capita per day in 2017-2018 [31,32]. This reduction is likely driven in large part by decreased red meat consumption, which is the single largest dietary contributor to GHGE. In the United States, red meat intake fell from 340 g/wk in 1999-2000 to 284 g/wk in 2015-2016 [33]. Although precise data are lacking, meat products are also major contributors to other environmental impact indicators, and their reduced consumption likely contributed to the overall decline [29]. Further research is warranted to identify the food groups most responsible for these improvements and to elucidate the pathways linking dietary shifts to environmental outcomes.

In contrast to adults aged 20-59 years, SDI-US scores among those aged ≥60 years declined over time. One possible explanation is differences in the motivations driving food choices. Motivation is a key driver of behavior change [34], and food choice motives may be critical to achieving sustainable and healthy diets [35]. A previous study reported that individuals in the “towards plant and organic foods” cluster, which is characterized by strong motives related to ethics, environmental concerns, local and traditional production, environmental avoidance, and healthy consumption, were more likely to be younger, female, and more educated [36]. In addition, adults in the “towards unhealthy foods” cluster, whose motives centered on price, convenience, and novelty, were typically older (≥65 years), male, and less educated [36].

Beyond motivational factors, health-related limitations such as chronic disease, poor oral health, and mobility constraints may cumulatively contribute to declining SDI-US scores among older adults. Chronic conditions can impair daily functioning, making food preparation more burdensome [37] and prompting a preference for ready-to-eat or processed foods, which are often less sustainable [38]. Poor oral health, including tooth loss and difficulty chewing, can increase reliance on processed meats [39]. Mobility limitations may reduce access to outlets that offer affordable, fresh foods [40]. These challenges may be further exacerbated by societal changes, such as the expansion of the convenience food market and an increasing number of single-person households. Further research should examine the interplay of personal, environmental, and structural factors influencing sustainable dietary patterns in older populations.

The decline in the economic indicator is particularly noteworthy, as it may reflect broader economic challenges during the study period. This pattern could be partly attributed to rising food prices driven by inflation, stagnating income levels, and persistent income inequality [41,42]. Older adults are especially vulnerable to poor nutrition and food insecurity when faced with financial constraints [43], suggesting that economic barriers may have contributed to the downward trend in SDI-US among those aged ≥60 years. Addressing these challenges will require targeted policy measures, including strengthening food assistance programs such as the Supplemental Nutrition Assistance Program [44], improving access to affordable and nutritious foods, and monitoring regional disparities in food costs and income.

This study has several limitations. First, the SDI-US may be subject to the inherent subjectivity of its metrics and indicator selection, the potential omission of relevant dimensions and indicators, and inconsistencies within the environmental impacts database. Additionally, several composite indices for multidimensional sustainable dietary patterns [45], including the SDI-US [5-9], have been criticized for potential trade-offs when combining subcomponents, the concealment of important decisions regarding the inclusion and representation of sub-indices, and the indirect relationships between sub-indices and the main variable of interest [46]. Nonetheless, the use of the SDI-US remains reasonable because (1) no single indicator can adequately capture the multidimensional nature of sustainable diets [47]; (2) emphasizing multidimensionality is valuable for advancing public, research, and policy progress toward more sustainable dietary practices [30]; (3) the SDI-US was constructed using clear, context-specific criteria for indicator selection based on the availability of individual-level dietary data in the United States [5], thereby minimizing subjective indicator choice and weighting methods [48,49]; and (4) the SDI-US has undergone multiple forms of validation [5]. Even so, further refinement of the SDI-US and caution in interpreting its results are warranted. Second, although the environmental footprint system boundaries in this study encompassed all pre-retail stages of the food system [29], we were unable to evaluate the relative contribution of each stage to the overall environmental burden, nor fully account for the impacts of ultra-processed food consumption, which are largely attributable to processing and packaging stages. Expanding footprint assessments to include post-retail stages represents an important area for future research. Third, self-reported assessments, including the 24-hour dietary recall, are inherently prone to measurement error and underreporting [24,25]. However, such biases may have been mitigated through the use of validated instruments, standardized NHANES data collection protocols [50] and energy-adjusted nutrient intake during the analysis [23,25]. Fourth, because NHANES employs a cross-sectional design, we cannot determine which changes in demographic or lifestyle factors contributed to the observed trends, nor how they did so. Fifth, while the economic sub-index accounts for food expenditures, including subsidies, it may not fully capture affordability due to regional variations in cost of living. Sixth, the negative scoring of socio-cultural indicators assumes that home-prepared meals are inherently more sustainable. This assumption may not hold in contemporary contexts—particularly for working adults and those living alone—where time constraints, convenience, and accessibility strongly influence food choices. Consequently, our approach may oversimplify complex eating behaviors and introduce cultural or contextual bias into both the total SDI-US and the socio-cultural sub-index. Seventh, indices such as MAR and SDI-US currently lack established clinical thresholds for meaningful change, unlike traditional biomarkers. Further research is needed to define what constitutes a meaningful change for population-level monitoring of dietary sustainability and for policy evaluation. Lastly, this study focuses exclusively on United States adults and may not be generalizable to adolescents or children.

The present study also has notable strengths. First, to our knowledge, it is the first to assess dietary sustainability among United States adults using a holistic approach that incorporates all 4 FAO-recommended dimensions—nutritional, environmental, economic, and socio-cultural. Second, NHANES is uniquely suited to this analysis, as it is the only survey that provides the comprehensive data required to calculate both the total SDI-US and its individual components.

In conclusion, despite growing public and scientific interest in sustainable diets, overall SDI-US scores remained low and largely unchanged from 2007 to 2018 in a nationally representative sample of United States adults. The 99th percentile score of 18.5 out of 20 suggests that the vast majority of United States adults aged ≥20 years could improve their dietary patterns to better align with sustainability principles. While environmental indicators improved over time, other indicators remained unchanged or worsened. Notably, there was a significant decline in SDI-US among older adults (≥60 years), indicating a deterioration in dietary sustainability within this group. Nevertheless, older adults continued to score higher than younger adults (20-39 years). These findings highlight the need for continued monitoring to identify strategies that effectively improve adherence to sustainable dietary patterns, with careful attention to interactions across all sustainability dimensions—not only in older populations but also among younger adults. Future research should also assess the applicability of the SDI-US across diverse ethnic and cultural groups with markedly different dietary patterns, enabling cross-cultural comparisons of sustainable dietary practices. Additionally, examining public understanding of sustainable diets, along with the motivators and barriers to adherence, will be essential for developing targeted communication strategies that promote meaningful and lasting behavior change.

## Figures and Tables

**Figure f2-epih-47-e2025045:**
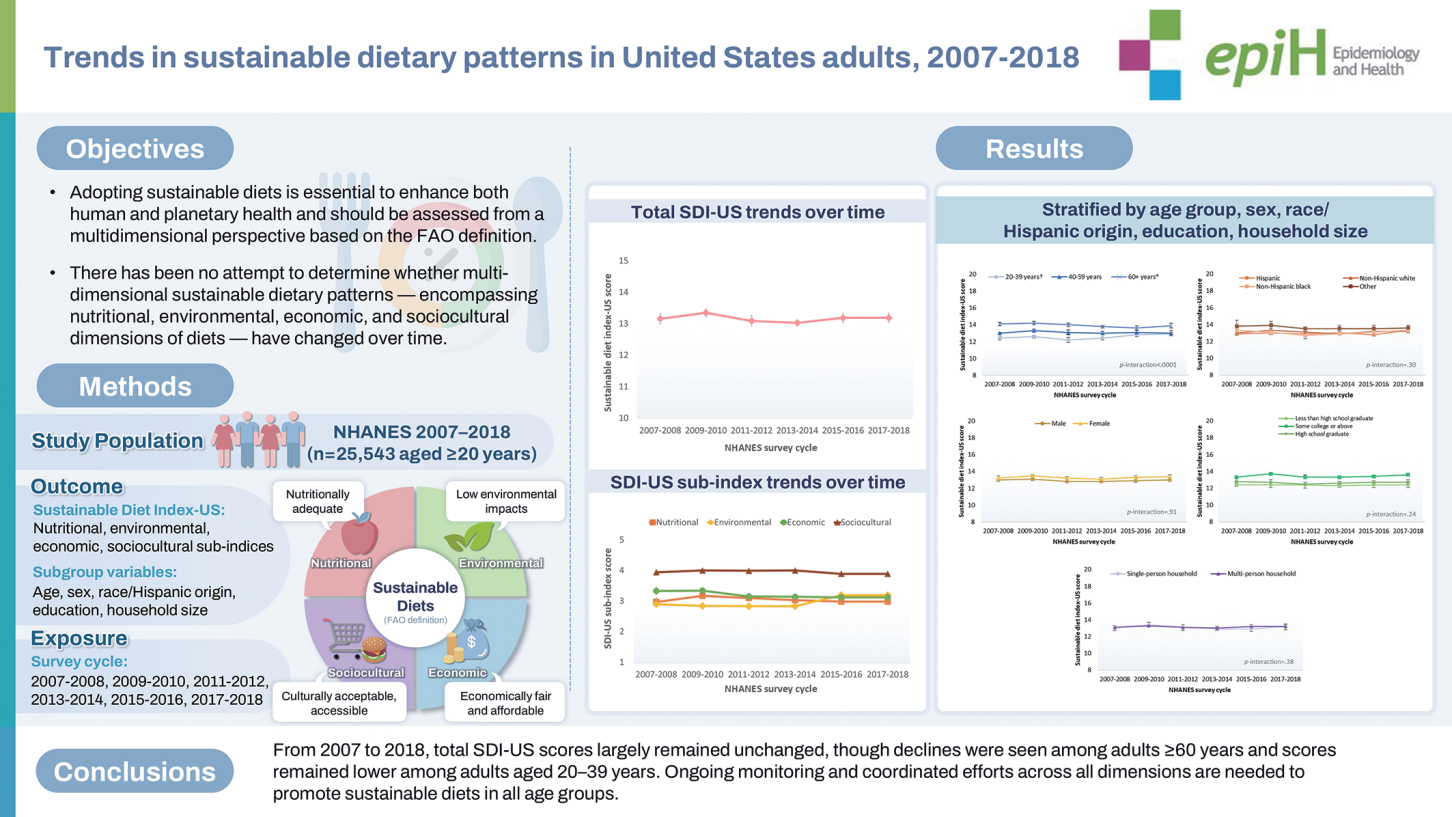


**Table 1.** Demographic characteristics by NHANES cycle, US adults, 2007-2018 (n=25,543)

Characteristics	No. of participants (weighted %)^1^					
	2007-2008 (n=3,996)	2009-2010 (n=4,887)	2011-2012 (n=4,120)	2013-2014 (n=4,408)	2015-2016 (n=4,212)	2017-2018 (n=3,920)
Age (yr)						
20-39	1,230 (36.1)	1,581 (35.6)	1,466 (35.6)	1,470 (34.7)	1,374 (34.7)	1,143 (35.1)
40-59	1,278 (39.4)	1,687 (39.6)	1,380 (38.6)	1,531 (37.5)	1,437 (37.5)	1,266 (35.2)
≥60	1,488 (24.6)	1,619 (24.8)	1,274 (25.8)	1,407 (27.7)	1,401 (27.8)	1,511 (29.7)
Sex						
Male	1,988 (47.3)	2,414 (48.9)	2,079 (49.6)	2,140 (49.6)	2,075 (49.0)	1,944 (49.1)
Female	2,008 (52.7)	2,473 (51.1)	2,041 (50.4)	2,268 (50.4)	2,137 (51.0)	1,976 (50.9)
Race/Hispanic origin						
Hispanic	1,084 (12.7)	1,304 (12.5)	797 (13.8)	944 (14.2)	1,277 (14.4)	843 (14.9)
Non-Hispanic White	2,006 (72.5)	2,503 (71.3)	1,660 (68.2)	2,024 (67.0)	1,505 (66.4)	1,498 (64.2)
Non-Hispanic Black	763 (10.0)	831 (10.5)	1,036 (10.7)	854 (10.7)	854 (10.1)	867 (10.4)
Other^2^	143 (4.7)	249 (5.8)	627 (7.4)	586 (8.1)	576 (9.1)	712 (10.5)
Education level						
Less than high school graduate	1,211 (19.6)	1,338 (18.0)	889 (15.0)	863 (14.3)	922 (12.8)	700 (10.1)
High school graduate or GED	961 (25.1)	1,115 (22.4)	862 (19.8)	1,000 (22.3)	947 (21.4)	963 (28.9)
Some college or above	1,824 (55.4)	2,434 (59.6)	2,369 (65.2)	2,545 (63.4)	2,343 (65.8)	2,257 (61.0)
Household size						
Single-person household	582 (14.1)	710 (14.3)	607 (13.4)	633 (14.2)	614 (14.2)	582 (12.9)
Multi-person household	3,414 (85.9)	4,177 (85.7)	3,513 (86.6)	3,775 (85.8)	3,598 (85.8)	3,338 (87.1)

NHANES, National Health and Nutrition Examination Survey; GED, general equivalency diploma.

1 Sample sizes are unweighted and all other estimates are weighted (dietary Day 1 weights).

2 “Other” includes race/Hispanic origin other than non-Hispanic White, non-Hispanic Black, and Hispanic, including multiracial.

**Table 2.** Trends in SDI-US and sub-index scores by NHANES cycle in US adults, 2007-2018

Variables	2007-2008 (n=3,996)	2009-2010 (n=4,887)	2011-2012 (n=4,120)	2013-2014 (n=4,408)	2015-2016 (n=4,212)	2017-2018 (n=3,920)	p-value for trend^1^	
							Linear	Quadratic
Unadjusted model								
Total SDI-US score	13.0 (12.9, 13.2)	13.3 (13.1, 13.4)	13.1 (12.9, 13.2)	13.0 (12.9, 13.2)	13.2 (13.0, 13.4)	13.3 (13.1, 13.5)	0.20	0.39
Sub-index scores								
Nutritional	2.9 (2.8, 3.1)	3.2 (3.1, 3.2)	3.1 (3.0, 3.2)	3.1 (3.0, 3.1)	3.0 (2.9, 3.1)	3.0 (2.9, 3.1)	0.80	0.02
Environmental	2.9 (2.7, 3.0)	2.8 (2.7, 2.8)	2.8 (2.7, 2.8)	2.8 (2.7, 2.8)	3.1 (3.1, 3.2)	3.2 (3.2, 3.3)	<0.001	<0.001
Economic	3.3 (3.2, 3.4)	3.3 (3.2, 3.4)	3.2 (3.0, 3.3)	3.1 (3.1, 3.2)	3.1 (3.0, 3.3)	3.1 (3.1, 3.2)	0.01	0.32
Sociocultural	3.9 (3.9, 4.0)	4.0 (3.9, 4.0)	4.0 (3.9, 4.0)	4.0 (4.0, 4.1)	3.9 (3.8, 4.0)	3.9 (3.8, 4.0)	0.12	0.01
Multivariable-adjusted model^2^								
Total SDI-US score	13.1 (12.9, 13.3)	13.3 (13.2, 13.4)	13.0 (12.9, 13.2)	13.0 (12.9, 13.1)	13.1 (13.0, 13.3)	13.2 (13.1, 13.4)	0.92	0.13
Sub-index scores								
Nutritional	3.0 (2.8, 3.1)	3.2 (3.1, 3.2)	3.1 (3.0, 3.2)	3.1 (3.0, 3.1)	3.0 (2.9, 3.1)	3.1 (2.9, 3.1)	0.20	0.04
Environmental	2.8 (2.7, 3.0)	2.8 (2.7, 2.8)	2.8 (2.7, 2.8)	2.8 (2.7, 2.8)	3.1 (3.1, 3.2)	3.2 (3.2, 3.3)	<0.001	<0.001
Economic	3.3 (3.2, 3.4)	3.3 (3.2, 3.4)	3.1 (3.0, 3.2)	3.1 (3.1, 3.2)	3.1 (3.0, 3.2)	3.1 (3.1, 3.2)	<0.001	0.10
Sociocultural	3.9 (3.9, 4.0)	4.0 (4.0, 4.1)	4.0 (4.0, 4.0)	4.0 (4.0, 4.1)	3.9 (3.8, 4.0)	3.9 (3.8, 4.0)	0.02	0.01

Values are presented as weighted mean (95% confidence interval); Using the linear regression model. NHANES, National Health and Nutrition Examination Survey; SDI-US, sustainable diet index for United States.

1 Estimated with the survey cycles modeled as an orthogonal polynomial.

2 Adjusted for age (categorical: 20-39, 40-59, or ≥60 years), sex (dichotomous: male or female), race/Hispanic origin (categorical: total Hispanic, non-Hispanic White, non-Hispanic Black, or other), education level (categorical: less than high school graduate, high school graduate or equivalent, some college or above), and household size (continuous) and weighted (dietary Day 1 sample weights) in the linear regression model.

**Table 3.** Trends in nutritional quality, diet-related environmental impacts, food affordability, and food practices by NHANES cycle, US adults, 2007-2018

Multivariable-adjusted model^1^	2007-2008 (n=3,996)	2009-2010 (n=4,887)	2011-2012 (n=4,120)	2013-2014 (n=4,408)	2015-2016 (n=4,212)	2017-2018 (n=3,920)	p-value for trend^2^	
							Linear	Quadratic
Nutritional								
NRF9.3 index	17.6±0.9	19.7±0.5	18.4±0.7	18.1±0.6	17.3±0.4	17.2±0.8	0.12	0.19
Mean nutrient adequacy ratio	69.9±0.7	71.9±0.2	71.8±0.4	70.9±0.3	70.5±0.4	70.3±0.4	0.49	0.01
Environmental								
Fresh water use (L)	378±12	397±9	410±7	422±9	357±11	328±9	<0.001	<0.001
Stress-weighted water use (L)	13,158±442	13,812±251	13,976±247	14,111±271	11,934±313	11,068±347	<0.001	<0.001
Acidifying emissions (g SO_2_eq)	25.0±0.7	26.1±0.5	25.6±0.7	25.5±0.4	20.0±0.6	19.0±0.4	<0.001	<0.001
Eutrophying emissions (g PO_4_^3-^eq)	19.7±0.7	20.4±0.4	19.4±0.6	20.0±0.6	15.9±0.6	15.0±0.5	<0.001	<0.001
GHGE (kg CO_2_eq)	4.4±0.2	4.6±0.1	4.5±0.2	4.3±0.1	3.8±0.2	3.7±0.1	<0.001	0.03
Land use (m^2^)	10.2±0.5	10.7±0.4	10.4±0.5	9.4±0.3	9.2±0.5	8.9±0.4	0.003	0.40
Economic								
Share of budget to food (%)	21.4±1.1	22.8±0.8	26.7±1.8	24.5±1.0	28.5±1.4	26.4±0.8	<0.001	0.12
Socio-cultural								
Frequency of meals from a fast-food or pizza place (times during the last 7 day)	1.7±0.1	1.5±0.04	1.7±0.1	1.7±0.1	1.7±0.1	1.7±0.1	0.15	0.20
Frequency of ready-to-eat products (times during the last 30 day)	1.9±0.1	1.5±0.1	1.7±0.1	2.2±0.2	2.2±0.1	2.2±0.2	0.001	0.30
Frequency of frozen meals and pizza (times during the last 30 day)	2.6±0.2	2.7±0.2	2.9±0.2	2.9±0.3	2.6±0.1	2.2±0.1	0.14	0.02

Values are presented as weighted mean±standard error; Using the linear regression model. NHANES, National Health and Nutrition Examination Survey; NRF9.3, Nutrient Rich Foods 9.3; GHGE, greenhouse gas emissions.

1 Adjusted for age (categorical: 20-39, 40-59, or ≥ 60 years), sex (dichotomous: male or female), race/Hispanic origin (categorical: total Hispanic, non-Hispanic White, non-Hispanic Black, or other), education level (categorical: less than high school graduate, high school graduate or equivalent, some college or above), and household size (continuous) and weighted (dietary Day 1 sample weights) in the linear regression model.

2 Estimated with the survey cycles modeled as an orthogonal polynomial.

## Supplementary materials

Supplementary Material 1. NHANES data collection procedures

Supplementary Material 2. Study participants flow chart, National Health and Nutrition Examination Survey (NHANES) adults aged 20 years and older, 2007-2018 (n=25,543)

Supplementary Material 3. Description of selected indicators and calculation of the total sustainable diet index-US and sub-index scores

Supplementary Material 4. Worked examples of nutritional indicators calculation

Supplementary Material 5. Description of sensitivity analyses

Supplementary Material 6. Trends in food group consumption by NHANES cycle, US adults, 2007-2018 (n=25,543)

Supplementary Material 7. Comparison of the sustainable diet index-US and modified sustainable diet index-US, US adults, NHANES 2007-2018 (n=25,543)
