# Supplementary material for: Trends in sustainable dietary patterns in United States adults, 2007-2018
Source: Epidemiol Health. 2025 Aug 18;47:e2025045. doi: 10.4178/epih.e2025045 (PMC12673291; doi:10.4178/epih.e2025045)
Supplement: Supplementary Material 1. — NHANES data collection procedures [file epih-47-e2025045-Supplementary-1.docx]

**Supplementary Material 1. NHANES data collection procedures**

| Household interview  During the household interview, data were collected on demographics, consumer behaviors, and dietary behaviors and nutrition. Demographic details included age, sex, race and Hispanic origin, education level (highest degree or level of school completed), and annual household and family income. Information related to consumer behavior and diet practices covered topics such as food expenditures, the frequency of meals prepared away from home, the frequency of meals from fast-food or pizza places, and the use of ready-to-eat meals purchased from stores.  Physical examination  During the MEC examination, a 24-hour dietary recall interview was conducted, alongside the collection of biological specimens, including blood. The dietary interview was carried out by trained dieticians to collect detailed dietary information from participants. The 24-hour dietary recall interview involved documenting the description, quantity, and time and place of eating of all foods, beverages, and water consumed in the past 24-hours (from midnight to midnight). Participants reported the amounts of foods and beverages consumed with help from a standard set of measuring tools such as glasses, bowls, mugs, bottles, household spoons, and other measuring instruments. The Automated Multiple Pass Method was used to ensure a complete and accurate food recall. The energy, nutrients, and food components of all reported foods and beverages in NHANES 2007-2018 were derived from the United States Department of Agriculture’s Food and Nutrient Database for Dietary Studies [1].  Serum levels of 25(OH)D_2_, 25(OH)D_3_, and C3 epimer of 25(OH)D_3_ were measured using a fully standardized and validated liquid chromatography-tandem mass spectrometry (LC-MS/MS) method [2]. In this study, total serum 25(OH)D (nmol/L) (25(OH)D_3_ + 25(OH)D_2_) was used.  US)  **References**  1. U.S. Department of Agriculture, Agricultural Research Service. 2018. USDA Food and Nutrient Database for Dietary Studies, 2015-2016 Factsheet. Food Surveys Research Group Home Page, Available online: <https://www.ars.usda.gov/ARSUserFiles/80400530/pdf/fndds/FNDDS_2015_2016_factsheet.pdf> (accessed June 1, 2021).  2. National Center for Health Statistics. National Health and Nutrition Examination Survey, 2015–2016 data documentation, codebook, and frequencies: Vitamin D. 2021. Available online: <https://wwwn.cdc.gov/nchs/nhanes/2015-2016/VID_I.htm> (accessed June 21, 2022). |
| --- |
