# Supplementary material for: Trends in sustainable dietary patterns in United States adults, 2007-2018
Source: Epidemiol Health. 2025 Aug 18;47:e2025045. doi: 10.4178/epih.e2025045 (PMC12673291; doi:10.4178/epih.e2025045)
Supplement: Supplementary Material 2. — Study participants flow chart, National Health and Nutrition Examination Survey (NHANES) adults aged 20 years and older, 2007-2018 (n=25,543) [file epih-47-e2025045-Supplementary-2.docx]

**Supplementary Material 2. Study participants flow chart, National Health and Nutrition Examination Survey (NHANES) adults aged 20 years and older, 2007-2018 (n=25,543)**

≥20 years of age in

NHANES 2007-2018

(n=34,770)

Pregnant or lactating women

(n=580)

(n=17,135)

Excluding pregnant or lactating women aged 20-44 years

(n=34,190)

Incomplete day one 24HR

(n=3944)

Missing on serum 25(OH)D

(n=2058)

Valid day one 24HR & serum 25(OH)D

(n=28,188)

Missing information on SDI-US calculation and effect modifiers (n=2645)

(n=17,135)

Final analytic sample

(n=25,543)
