# Supplementary material for: Trends in sustainable dietary patterns in United States adults, 2007-2018
Source: Epidemiol Health. 2025 Aug 18;47:e2025045. doi: 10.4178/epih.e2025045 (PMC12673291; doi:10.4178/epih.e2025045)
Supplement: Supplementary Material 3. — Description of selected indicators and calculation of the total sustainable diet index-US and sub-index scores [file epih-47-e2025045-Supplementary-3.docx]

**Supplementary Material 3. Description of selected indicators and calculation of the total sustainable diet index-US and sub-index scores**

|  |  |  |  | **SDI-US** |  |
| --- | --- | --- | --- | --- | --- |
| Sub-index | Measure^a^ | Relationship with sustainable diets^a^ | Indicators in the sub-index | Points allocating | Assessment |
| Nutritional (/5) | Dietary diversity index | Diet diversity with nutrient adequacy is essential to avoid malnutrition and negative health outcomes. | 1-1) Nutrient-Rich Foods9.3 Index^b^ | 1-point: ind≤4.1  2-point: 4.1<ind≤10.6  3-point: 10.6<ind≤18.2  4-point: 18.2<ind≤30.5  5-point: ind>30.5 | Nutrition sub-index=  the sum of points from indicator 1-1 to 1-2 × 1/2 |
|  | Micronutrient deficiencies of vitamins and minerals |  | 1-2) Mean Nutrient Adequacy Ratio^c^ | 1-point: ind≤60.2  2-point: 60.2<ind≤68.1  3-point: 68.1<ind≤74.2  4-point: 74.2<ind≤80.5  5-point: ind>80.5 |  |
| Environmental (/5) | Water footprint | Clean water resource is becoming scarce in zones. | 2-1) Freshwater withdrawals (L) per serving food^d^ | 1-point: ind>549.9  2-point: 377.1<ind≤549.9  3-point: 263.7<ind≤377.1  4-point: 161.5<ind≤263.7  5-point: ind≤161.4 | Environment sub-index=  the sum of points from indicator 2-1 to 2-6 × 1/6 |
|  |  |  | 2-2) Stress-weighted water use (L) per serving food^d^ | 1-point: ind>18475  2-point: 12806<ind≤18475  3-point: 9079<ind≤12806  4-point: 5601<ind≤9079  5-point: ind≤5601 |  |
|  | Nitrogen footprint | Nitrogen balance is essential to avoid eutrophication  and harmful algae blooms. | 2-3) Acidifying emissions (g SO_2_eq, CML2 baseline) per serving food^d^ | 1-point: ind>34.4  2-point: 22.6<ind≤34.4  3-point: 15.4<ind≤22.6  4-point: 9.3<ind≤15.4  5-point: ind≤9.3 |  |
|  |  |  | 2-4) Eutrophying emissions (g PO_4_^3-^eq, CML2 Baseline) per serving food^d^ | 1-point: ind>28.0  2-point: 16.3<ind≤28.0  3-point: 10.2<ind≤16.3  4-point: 6.1<ind≤10.2  5-point: ind≤6.1 |  |
|  | Carbon footprint | Anthropogenic greenhouse gas emissions contribute to climate change. | 2-5) Greenhouse gas emissions (kg CO_2_eq, IPCC 2013 includes feedbacks) per serving food^d^ | 1-point: ind>5.8  2-point: 3.4<ind≤5.8  3-point: 2.2<ind≤3.4  4-point: 1.4<ind≤2.2  5-point: ind≤1.4 |  |
|  | Land use | The availability of arable land is limited; moreover, land use change impacts the biodiversity preservation. | 2-6) Land use (m^2^) per serving food^d^ | 1-point: ind>13.0  2-point: 5.9<ind≤13.0  3-point: 3.7<ind≤5.9  4-point: 2.1<ind≤3.7  5-point: ind≤2.1 |  |
| Economic (/5) | Affordability | Healthy diet should be available at affordable prices to all, specifically to low-income consumers. | 3) Proportion of income devoted to diet | 1-point: ind>34.1  2-point: 20.0<ind≤34.1  3-point: 13.3<ind≤20.0  4-point: 9.0<ind≤13.3  5-point: ind≤9.0 | Economic sub-index=  the sum of points × 1 |
| Sociocultural (/5) | Ready-made products | The use of ready-made products minimizes cooking activities and thus limit the opportunity for social exchange, cultural  heritage preservation and trying diverse recipes. | 4-1) Frequency of meals not home prepared and from a fast-food or pizza place | 1-point: ind>4  2-point: 2<ind≤4  3-point: 1<ind≤2  4-point: 0<ind≤1  5-point: ind=0 | Sociocultural sub-index=  the sum of points from indicator 4-1 to 4-3 × 1/3 |
|  |  |  | 4-2) Frequency of ready-to-eat products | 1-point: ind>5  2-point: 3<ind≤5  3-point: 1<ind≤3  4-point: 0<ind≤1  5-point: ind=0 |  |
|  |  |  | 4-3) Frequency of frozen meals/pizza | 1-point: ind>7  2-point: 3<ind≤7  3-point: 2<ind≤3  4-point: 0<ind≤2  5-point: ind=0 |  |
| **Total SDI-US = nutritional + environmental + economic + sociocultural (range: 4-20)** | | | | | |

Abbreviation: SDI-US, sustainable diet index-US; ind, indicator

^a^ Source: Seconda L, Baudry J, Pointereau P, et al. Development and validation of an individual sustainable diet index in the NutriNet-Santé study cohort. *Br J Nutr* 2019;121:1166-77.

^b^ Sources: Fulgoni VL, 3rd, Keast DR, Drewnowski A. Development and validation of the nutrient-rich foods index: a tool to measure nutritional quality of foods; Drewnowski A. Defining nutrient density: development and validation of the nutrient rich foods index. *J Am Coll Nutr* 2009;28:421s-6s.

^c^ Source: Guthrie HA, Scheer JC. Nutritional adequacy of self-selected diets that satisfy the four food groups guide. *J Nutr Education* 1981;13:46-9.

^d^ Source: Source: Poore J, Nemecek T. 2018. Reducing food's environmental impacts through producers and consumers. Science 360:987-992 and Bryan T, Hicks A, Barrett B, et al. An environmental impact calculator for 24-h diet recalls. *Sustainability* 2019;11(23):686
