# Supplementary material for: Trends in sustainable dietary patterns in United States adults, 2007-2018
Source: Epidemiol Health. 2025 Aug 18;47:e2025045. doi: 10.4178/epih.e2025045 (PMC12673291; doi:10.4178/epih.e2025045)
Supplement: Supplementary Material 4. — Worked examples of nutritional indicators calculation [file epih-47-e2025045-Supplementary-4.docx]

**Supplementary Material 4**. Worked examples of nutritional indicators calculation

| NRF9.3 calculation   \|  \| Reference value \| Actual intake \| % daily value (DV) \| Sum of DVs \| \| --- \| --- \| --- \| --- \| --- \| \| **Nutrients to encourage** \|  \|  \|  \| 880 \| \| Protein \| 50 \| 100 \| 100/50*100 = 200 \| \| Fiber \| 50 \| 20 \| 20/50*100 = 40 \| \| Vitamin A \| 5000 \| 3000 \| 3000/5000*100 = 60 \| \| Vitamin C \| 60 \| 150 \| 150/60*100 = 250 \| \| Vitamin E \| 20 \| 10 \| 10/20*100 = 50 \| \| Iron \| 18 \| 18 \| 18/18*100 = 100 \| \| Calcium \| 1000 \| 500 \| 500/1000*100 = 50 \| \| Potassium \| 3500 \| 2800 \| 2800/3500*100 = 80 \| \| Magnesium \| 400 \| 200 \| 200/400*100 = 50 \| \| **Nutrients to limit** \|  \|  \|  \| 290 \| \| Saturated fat \| 20 \| 25 \| 25/20*100 = 125 \| \| Sodium \| 2400 \| 3000 \| 3000/2400*100 = 125 \| \| Added sugar \| 50 \| 20 \| 20/50*100 = 40 \|   NRF 9.3 = 880-290 = 590  NRF9.3 per 100 kcal = 590/2000*100 = 29.5  MAR calculation  If a female aged 25 years old consumed vitamin A, thiamin, vitamin B6, folate, vitamin C, and had serum vitamin D levels,   \|  \| Reference intake \| Actual intake \| NAR \| Capped at 1 \| \| --- \| --- \| --- \| --- \| --- \| \| **Vitamin A (µg RAE)** \| 700 \| 800 \| 700/800 = 1.14 \| 1 \| \| Thiamin (mg) \| 1.1 \| 0.8 \| 0.8/1.1 = 0.73 \|  \| \| Vitamin B6 (mg) \| 1.3 \| 1.5 \| 1.5/1.3 = 1.15 \| 1 \| \| Folate (µg DFE) \| 400 \| 320 \| 320/400 = 0.8 \|  \| \| Vitamin C (mg) \| 75 \| 90 \| 90/75 = 1.2 \| 1 \| \| Serum vitamin D (nmol/L) \| 50 \| 48 \| 48/50 = 0.96 \|  \|   Sum of NARs = 1+0.73+1+0.8+1+0.96 = 5.49  MAR = 5.49/6 = 0.91 |
| --- | --- | --- | --- | --- | --- | --- | --- | --- | --- | --- | --- | --- | --- | --- | --- | --- | --- | --- | --- | --- | --- | --- | --- | --- | --- | --- | --- | --- | --- | --- | --- | --- | --- | --- | --- | --- | --- | --- | --- | --- | --- | --- | --- | --- | --- | --- | --- | --- | --- | --- | --- | --- | --- | --- | --- | --- | --- | --- | --- | --- | --- | --- | --- | --- | --- | --- | --- | --- | --- | --- | --- | --- | --- | --- | --- | --- | --- | --- | --- | --- | --- | --- | --- | --- | --- | --- | --- | --- | --- | --- | --- | --- | --- | --- | --- | --- | --- | --- |
