# Supplementary material for: Trends in sustainable dietary patterns in United States adults, 2007-2018
Source: Epidemiol Health. 2025 Aug 18;47:e2025045. doi: 10.4178/epih.e2025045 (PMC12673291; doi:10.4178/epih.e2025045)
Supplement: Supplementary Material 5. — Description of sensitivity analyses [file epih-47-e2025045-Supplementary-5.docx]

**Supplementary Material 5. Description of sensitivity analyses**

| Sensitivity analyses  As sensitivity analyses, we repeated the trend analyses described as follows: 1) calculating the SDI-US using the healthy eating index (HEI)-2015 instead of the NRF9.3; 2) calculating the SDI-US using 5 environmental indicators after excluding one water-related indicator (freshwater use) to consider the possible effect of double counting on water in relation to the other indicators; 3) calculating the SDI-US by additionally including food security level in the economic sub-index to better represent food affordability; 4) the SDI-US by additionally including eating together with family or friends to the sociocultural sub-index because higher frequency of ready-to-eat meals may not necessarily indicate negative sociocultural practices related to social exchange or trying diverse recipes if people are eating together. However, this was tested only in NHANES 2007-2010 due to data availability. |
| --- |
