# Supplementary material for: Trends in sustainable dietary patterns in United States adults, 2007-2018
Source: Epidemiol Health. 2025 Aug 18;47:e2025045. doi: 10.4178/epih.e2025045 (PMC12673291; doi:10.4178/epih.e2025045)
Supplement: Supplementary Material 6. — Trends in food group consumption by NHANES cycle, US adults, 2007-2018 (n=25,543) [file epih-47-e2025045-Supplementary-6.docx]

**Supplementary Material 6. Trends in food group consumption by NHANES cycle, US adults, 2007-2018 (n=25,543)**

|  | *Weighted mean (SE)^a^* | | | | | | *P* value for trend^b^ | |
| --- | --- | --- | --- | --- | --- | --- | --- | --- |
| Food group | 2007-2008 (n=3996) | 2009-2010 (n=4887) | 2011-2012 (n=4120) | 2013-2014 (n=4408) | 2015-2016 (n=4212) | 2017-2018 (n=3920) | linear | quadratic |
| Whole grains (ounce eq/d) | 0.7 (0.1) | 0.8 (0.1) | 1.0 (0.1) | 0.9 (0.04) | 0.9 (0.1) | 0.8 (0.1) | 0.23 | **0.002** |
| Refined grains (ounce eq/d) | 5.7 (0.1) | 5.6 (0.1) | 5.6 (0.1) | 5.6 (0.1) | 5.4 (0.1) | 5.6 (0.1) | 0.05 | 0.45 |
| Total vegetables (cup eq/d) | 1.5 (0.05) | 1.6 (0.04) | 1.6 (0.1) | 1.5 (0.04) | 1.6 (0.05) | 1.5 (0.1) | 0.53 | 0.79 |
| Total fruit (cup eq/d) | 1.0 (0.1) | 1.1 (0.04) | 1.0 (0.04) | 0.9 (0.05) | 0.9 (0.1) | 0.8 (0.05) | **<.0001** | 0.55 |
| Total dairy (cup eq/d) | 1.5 (0.1) | 1.7 (0.1) | 1.6 (0.1) | 1.6 (0.1) | 1.5 (0.1) | 1.4 (0.1) | **<.001** | **0.0004** |
| Red meat (ounce eq/d) | 1.7 (0.1) | 1.7 (0.1) | 1.7 (0.1) | 1.5 (0.1) | 1.7 (0.1) | 1.6 (0.1) | 0.20 | 0.35 |
| Eggs (ounce eq/d) | 0.5 (0.03) | 0.5 (0.03) | 0.5 (0.03) | 0.6 (0.03) | 0.6 (0.03) | 0.6 (0.04) | **0.004** | 0.09 |
| Soybean products (ounce eq/d) | 0.1 (0.01) | 0.1 (0.01) | 0.1 (0.01) | 0.1 (0.01) | 0.1 (0.02) | 0.1 (0.02) | **0.03** | 0.31 |
| Nuts (ounce eq/d) | 0.6 (0.1) | 0.7 (0.1) | 0.8 (0.1) | 0.8 (0.1) | 0.8 (0.1) | 0.8 (0.1) | **0.01** | 0.12 |
| Added sugar (grams/d) | 16.0 (0.5) | 15.6 (0.3) | 16.5 (0.3) | 16.0 (0.3) | 15.0 (0.3) | 15.4 (0.4) | 0.15 | 0.36 |

Abbreviations: NHANES, National Health and Nutrition Examination Survey; SE, standard error.

^a^ Estimated mean and SE were obtained using the linear regression model.

^b^ *P* values for trends were estimated with the survey cycles modeled as an orthogonal polynomial.

^c^ Food group consumption was adjusted for age, sex, race/Hispanic origin, household size, and total energy intake and weighted (dietary Day 1 sample weights) in the linear regression model.
