# Supplementary material for: Trends in sustainable dietary patterns in United States adults, 2007-2018
Source: Epidemiol Health. 2025 Aug 18;47:e2025045. doi: 10.4178/epih.e2025045 (PMC12673291; doi:10.4178/epih.e2025045)
Supplement: Supplementary Material 7. — Comparison of the sustainable diet index-US and modified sustainable diet index-US, US adults, NHANES 2007-2018 (n=25,543) [file epih-47-e2025045-Supplementary-7.docx]

**Supplementary Material 7. Comparison of the sustainable diet index-US and modified sustainable diet index-US, US adults, NHANES 2007-2018 (n=25,543)**

|  | *Weighted mean (95% CI) ^a^* |  |  |  |  |  | *P* value for trend ^b^ |  | Correlation ^c^ | Weighted kappa ^d^ |
| --- | --- | --- | --- | --- | --- | --- | --- | --- | --- | --- |
|  | 2007-2008 (n=3996) | 2009-2010 (n=4887) | 2011-2012 (n=4120) | 2013-2014 (n=4408) | 2015-2016 (n=4212) | 2017-2018 (n=3920) | linear | quadratic |  |  |
| **Original SDI-US** | 13.1  (12.9-13.3) | 13.3  (13.2-13.4) | 13.0  (12.9-13.2) | 13.0  (12.9-13.1) | 13.1  (13.0-13.3) | 13.2  (13.1-13.4) | 0.92 | 0.13 |  |  |
| **Modified version** |  |  |  |  |  |  |  |  |  |  |
| mSDI-US-1  NRF9.3 → HEI-2015 | 13.1  (13.0-13.2) | 13.3  (13.2-13.4) | 13.1  (12.9-13.2) | 13.0  (12.9-13.1) | 13.1  (13.0-13.3) | 13.2  (13.0-13.4) | 0.97 | 0.30 | 0.97 | 0.84 |
| mSDI-US-2  Excluded freshwater use | 13.1  (12.9-13.3) | 13.3  (13.2-13.4) | 13.0  (12.9-13.2) | 13.0  (12.9-13.1) | 13.1  (13.0-13.3) | 13.2  (13.1-13.4) | 0.82 | 0.15 | 0.998 | 0.98 |
| mSDI-US-3  Included food security level | 13.1  (12.9-13.3) | 13.3  (13.2-13.4) | 13.0  (12.9-13.2) | 13.0  (12.9-13.1) | 13.1  (13.0-13.3) | 13.2  (13.0-13.4) | 0.92 | 0.15 | 0.998 | 0.98 |
| mSDI-US-4  Included eating together | 12.7  (12.6-12.9) | 12.9  (12.8-13.1) | NA | NA | NA | NA | NA | NA | 0.988 | 0.91 |

Abbreviations: NHANES, National Health and Nutrition Examination Survey; CI, confidence interval; SDI-US, sustainable diet index-US; mSDI-US, modified sustainable diet index-US; NRF9.3, Nutrient-Rich Foods9.3; HEI, Healthy Eating Index.

Note: mSDI-US-1 was calculated using the Healthy Eating Index-2015 instead of using Nutrient-Rich Foods9.3 index because better nutritional composition does not necessarily translate into overall healthier diet; mSDI-US-2 was calculated using 5 environmental indicators after excluding one water-related indicator (freshwater use) to consider the possible effect of double counting on water in relation to the other indicators; mSDI-US-3 was calculated by additionally including food security level to the economic sub-index to better represent food affordability; mSDI-US-4 was calculated by additionally including eating together with family or friends to the sociocultural sub-index because higher frequency of ready-to-eat meals may not necessarily indicate negative sociocultural practices related to social exchange or trying diverse recipes if people are eating together. However, this was tested only in NHANES 2007-2010 due to data availability.

^a^ Estimated mean and 95% CI were adjusted for age (categorical: 20-39, 40-59, or ≥ 60 y), sex (dichotomous: male or female), race/Hispanic origin (categorical: Total Hispanic, Non-Hispanic White, Non-Hispanic Black, or Other), education level (categorical: less than high school graduate, high school graduate or equivalent, some college or above), and household size (continuous) and weighted (dietary Day 1 sample weights) in the linear regression model.

^b^ *P* values for trends were estimated with the survey cycles modeled as an orthogonal polynomial.

^c^ The Pearson correlation coefficients of the original SDI-US with the modified SDI-US were estimated using the linear regression model.

^d^ The weighted Kappa coefficients were obtained from a cross-classification analysis to compare the agreement of classification into quintiles between the original SDI-US and the modified SDI-US
